# Genetics and Genomic Medicine in Colombia

**DOI:** 10.1002/mgg3.139

**Published:** 2015-03-05

**Authors:** Mauricio De Castro, Carlos Martín Restrepo

**Affiliations:** 1 National Human Genome Research Institute, National Institutes of HealthBethesda, Maryland; 2 Unidad de Genética, DCB, Universidad Del RosarioBogotá, Colombia

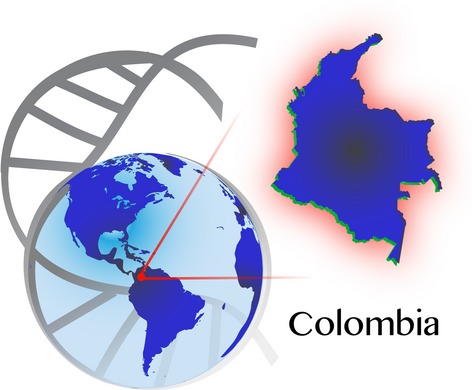



## Colombia: A Diverse Country

**Figure 1:**
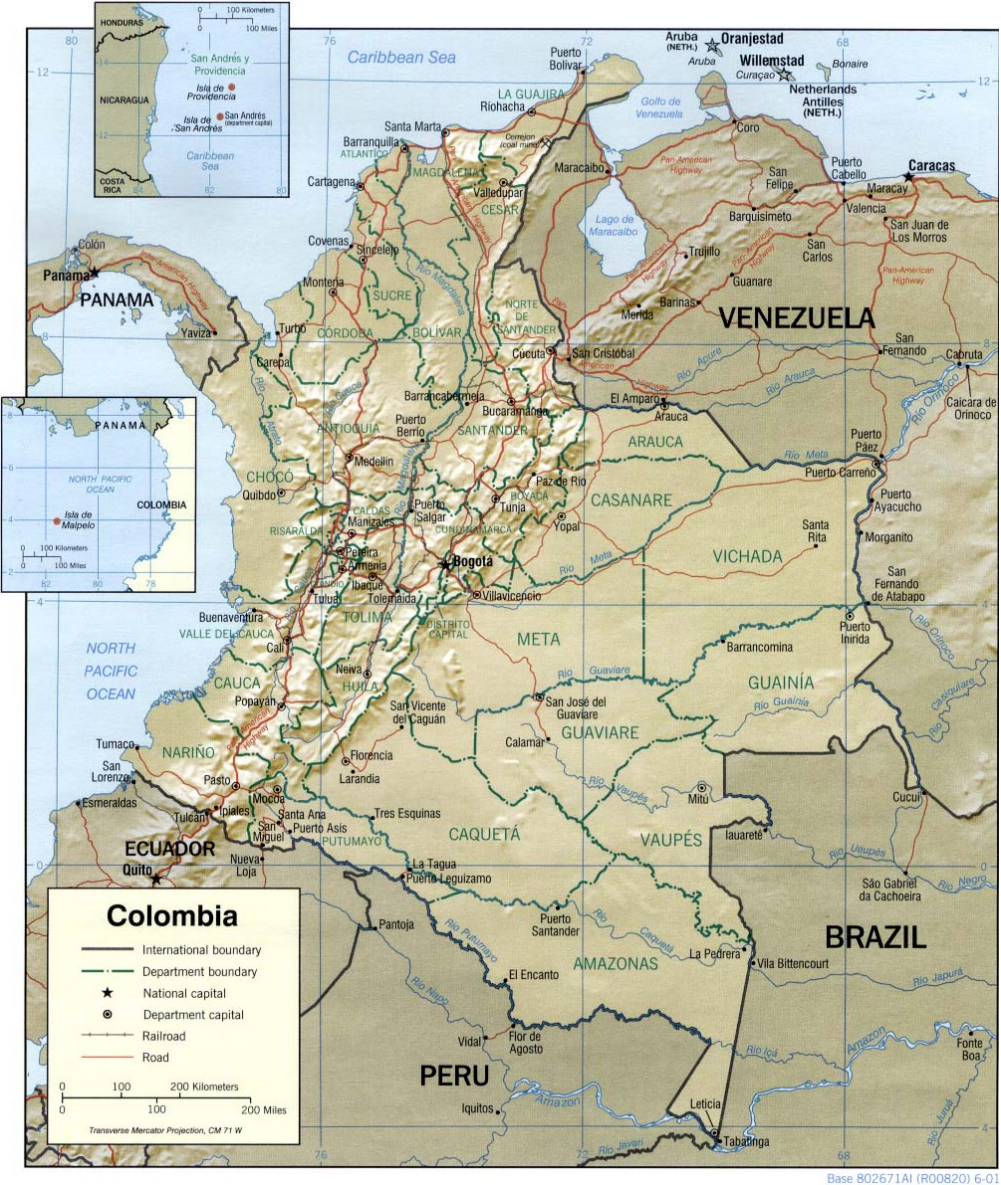
Political map of Colombia (*Source:* Wikimedia).

Colombia is a country located in the northwest corner of South America (Fig.1). Initially founded in 1717 as the Viceroyalty of New Grenada, it underwent many transitions in its government and territory after winning its independence from Spain in 1819, finally becoming the Republic of Colombia in 1886 (CIA, World Fact Book: https://www.cia.gov/library/publications/the-world-factbook/geos/co.html ).

Known officially as the Republic of Colombia, it shares borders with Venezuela and Brazil to the east; with Peru and Ecuador to the south; and with Panama to the Northwest; it also shares maritime borders with Nicaragua, Honduras, Jamaica, Dominican Republic, Haiti, and Costa Rica. Colombia is in a unique position in South America, possessing coasts on both Oceans; it borders the Caribbean Sea between Venezuela and Panama and the Pacific Ocean between Ecuador and Panama (CIA, World Fact Book).

It has a total land area of 1,138,910 square km, which makes it slightly less than twice the size of Texas. Its gross domestic product (GDP) was 378.4 billion in 2013. Colombia's growth was 4.7% in 2013, which was above the regional average of 3.7%. The World Bank considers Colombia a middle power and is part of the CIVETs group of six leading emergent markets (Schulz 2010). Colombia is also one of 17 megadiverse countries in the world. It is considered the most biodiverse by square kilometer (Declaración de Cancún: http://www.inecc.gob.mx/descargas/ai/con199328.pdf) and has the largest number of endemisms (species not found anywhere else) (Convention on Biological Diversity: http://www.cbd.int/countries/?country=co). Colombia hosts 10% of all species in the world, including 10% of all mammals, 14% of amphibian species, and 20% of birds; it has 32 terrestrial biomes and 314 types of ecosystems (SIB Colombia: http://www.sibcolombia.net/web/sib/cifras).

Colombia has suffered from asymmetric low-intensity armed conflict for nearly 60 years; the conflict has decreased considerably since the year 2000 owing to governmental efforts to curb the violence (CIA, World Fact Book). Its life expectancy is 74, on par with the regional standard.

## Population Diversity

Colombia is the third most populous country in Latin America with 47 million people as of 2014 (Departamento Administrativo Nacional de Estadística, DANE: https://www.dane.gov.co). The population of this ethnically diverse country is composed of three major groups: the descendants of its indigenous people (Amerindians), European immigrants (mostly Spanish), and Africans originally brought as slaves. Later waves of immigration in the 20th century would bring individuals from the Middle East and Romani populations, although these represent a small minority. There were also waves of German immigrants around both world wars.

Colombia is second only to Brazil in terms of number of indigenous tribes. Its indigenous peoples, composed of over 80 tribes, speak over 66 different languages; each tribe has its own values, believes, customs and religious, and artistic expressions (Aristizabal 2000). Among the indigenous peoples, two tribes currently count among their members over 100,000 individuals; the Nasa and Wayuu. Many of the tribes count less than 100 individuals with some on the verge of extinction such as the Taiwano, Pisamira, Makaguaje, and Bara (Aristizabal 2000). The Amerindian population is located in the plains, Amazonian jungle and in some regions of the Colombian Andes.

The Afro-Colombian population is descendant of slaves brought from Senegal, Ivory Coast and Mali during the early 16th century and for the most part settled along the Pacific coast of the country with a minority settling in the Caribbean coast and Islands. Raizal is the name given to individuals of a separate ethnic Afro-Caribbean group that populates the islands north of Colombia (San Andres y Providencia); they speak English Creole and have distinct religious beliefs (Pasaporte Colombiano: https://pasaportecolombiano.wordpress.com/2007/12/29/documento-acerca-de-los-raizales-de-san-andres).

**Figure 2:**
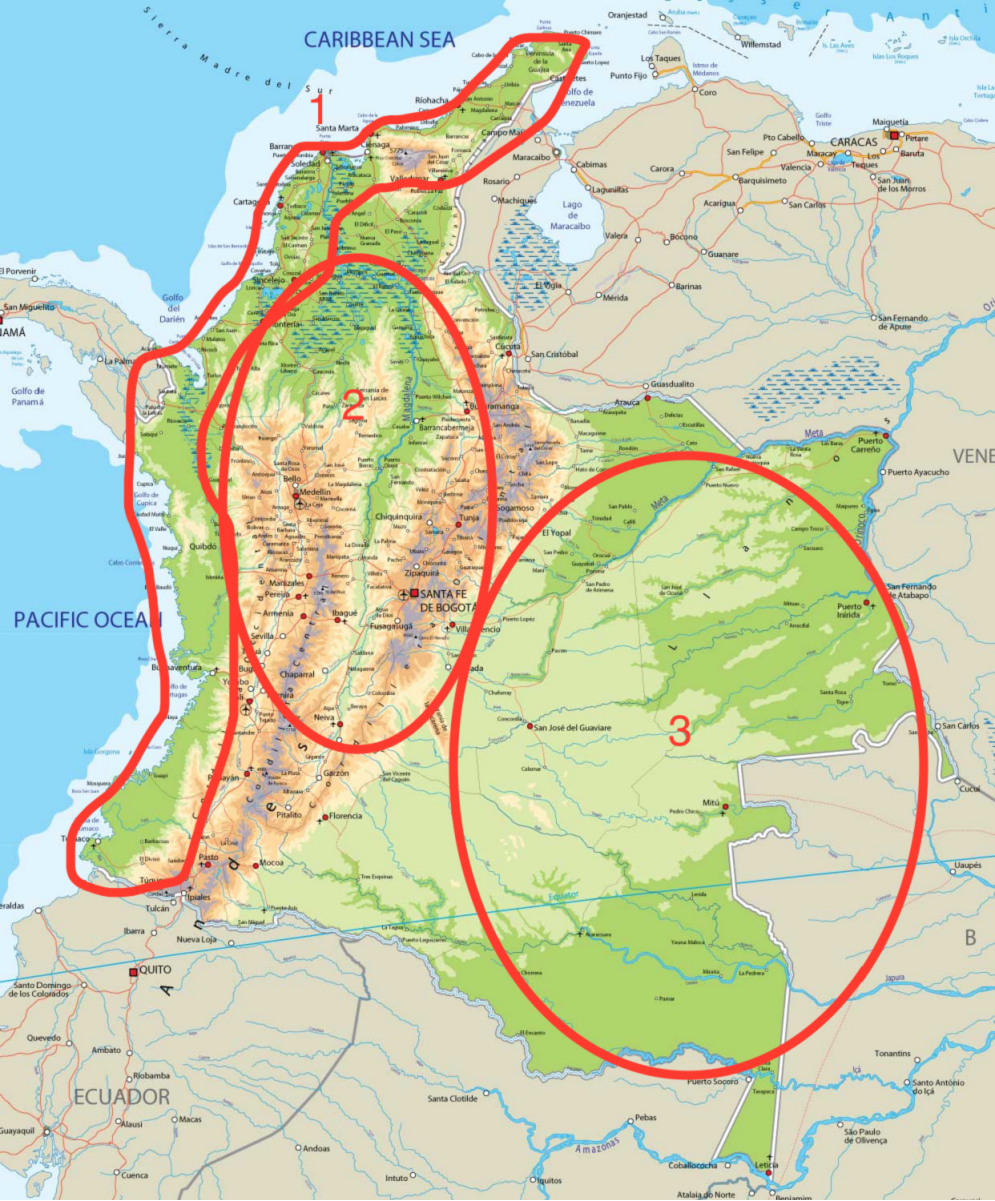
Physical map of Colombia showing a rough distribution of its populations. (1) Atlantic and Pacific coasts have the highest density of Afro-Colombian populations; (2) Andean highlands; (3) Amazonian region, with the highest density of Amerindian populations. Not pictured are the Islands of San Andres and Providencia (*Source:* Embassyworld.com).

According to the 2005 census, 49% of the population is mestizo (mixed European and Amerindian ancestry), 37% is of European ancestry (predominantly Spanish), 10% is of African ancestry, and 3.4% identify as Amerindian (DANE). Other minority populations of note are Lebanese, Palestinian, Chinese, Japanese, Romanians, and Jewish. The majority of the Colombian population is concentrated in the Andean highlands and the Caribbean coast, the nine eastern lowland departments, compromising 50% of the land area only account for ∽3% of the population. Figure2 shows a rough distribution of the different populations.

Genetic studies attempting to determine the degree of admixture in the country have shown that the population inhabiting the Pacific and Caribbean coast shows different degrees of admixture between African and mestizo descendants while Amerindian admixture is strongest to the Southwest and decreases toward the North portion of the country, where the Caucasian component is strongest (Yunis et al. 2013). Studies carried out within the Antioquia region showed that 94% of markers on the Y chromosome are of European descent, 5% are African, and 1% is Amerindian suggesting that most founders came from Europe, in particular southern Spain, with a fraction being of Sephardic Jewish Ancestry. In stark contrast to this, 90% of the mtDNA gene pool in this region is Amerindian, suggesting that the immigrant Spanish population mated in large numbers with the indigenous Amerindian women while the local men were largely eradicated as a result of disease, slave trade, and war (Carvajal-Carmona et al. 2000). A second corollary to this is that there was little to no immigration of Spanish women (Sanchez-Albornoz, 1987).

A separate study looking at mtDNA in three populations (mestizos, Afro-Colombians, and Amerindians) confirmed historical records supporting progressive settling of the Americas with successive waves of immigrants from the Asian continent, showing that Colombia served as an important bridge between Central and South America for these populations moving southward. This study also confirmed previous results regarding a drastic reduction in the native Amerindian populations presumably due to disease, war, and slavery with several local populations undergoing a massive bottleneck and some completely disappearing during the Spanish colonization. Although both mestizos and some of the local Amerindians currently show relatedness in mitochondrial haplotypes, the Afro-Colombian population shows a markedly distinct pattern, no doubt reflecting its separate African origin. (Instituto Colombiano de Cultura Hispánica).

## Healthcare in Colombia

After an overhaul to the Colombian national constitution in 1991, Law 100 of 1993 was created; the purpose of the law was to dramatically expand health coverage to the population, entitling all citizens, irrespective of their ability to pay, to comprehensive health benefits (Gideon and VILLAR URIBE 2009). In Colombia, citizens can participate in one of two regimens: the contributory regime, which covers workers who meet a threshold of minimum monthly income; and the subsidized regime, which covers the poor. Financing is carried out through payroll tax contributions (Gideon and Villar Uribe 2009). Benefit packages are tiered based on the regime individuals belong to, for the contributory regime, the POS (Plan Obligatorio de Salud) includes all levels of care, for the subsidized regime, the POSS (Plan Obligatorio de Salud Subsidiado), covers mostly low-complexity care and catastrophic illness. In Colombia, individuals are free to choose from a variety of insurers; ownership of these health-promoting entities (EPS, Entidad Promotora de Salud) can be public, private, or mixed. It is important to note that the Colombian Constitutional Court matched both benefit packages, POS and POSS, in 2009 (Judgment T-230/09 Corte Constitucional de Colombia: http://www.corteconstitucional.gov.co/relatoria/2009/T-230-09.htm).

Despite major efforts to expand healthcare coverage to all Colombian citizens (before 1993, health care coverage was 26%, in 2006 it was 96%), health care delivery continues to be disparate and the provision of services unequal. The quality of health care for the poor tends to fall behind accepted standards of care and there are concerns of pervasive, widespread corruption in the system (America Economia Magazine: http://www.worldcrunch.com/world-affairs/the-corruption-at-the-heart-of-colombia-039-s-health-care-system/colombia-health-care-corruption-privatization/c1s9688/#.VMK4ksaA3dm). It is a stark contrast: While Colombia has reached near-universal health insurance coverage and has grown as a destination for the health tourism industry with some of the best hospitals in Latin America (Ranking Revista America Economia: http://rankings.americaeconomia.com/mejores-clinicas-hospitales-2014/ranking/), some of the population has little or no access to adequate health care. In terms of quality and coverage, the rural areas in the non-Andean regions suffer the most.

In 2002, Colombia had 58,761 physicians, 23,950 nurses, and 33,951 dentists. In 2005, Colombia had 1.1 physicians per 1000 population as compared to an average of 1.5 for Latin America (Library of Congress, Colombia: http://lcweb2.loc.gov/frd/cs/cotoc.html). General government spending of health care in Colombia accounted for 20% of total government expenditure in 2005 (World Bank: http://www.worldbank.org/en/country/colombia). The burden of health care in Colombia is caused by chronic conditions with 75% of reports of morbidity and mortality related to ongoing, untreated medical conditions. The top five causes of morbidity in Colombia for women for all age groups were mental disorders, hypertensive disease, birth trauma, low birth weight, and glaucoma. (Cendex: http://cendex.javeriana.edu.co). In terms of reproductive health, the average fertility rate is 2.1, the rate being higher in rural areas and lower near urban centers (Encuesta Nacional de Demografia y Salud, 2010: http://www.profamilia.org.co/encuestas/Profamilia/Profamilia/index.php?option=com_content&view=article&id=62&Itemid=9). The average age at first birth is 21 years and the median interval between births is 48 months. Prenatal care is carried out in 97% of pregnancies and deliveries were carried out in a hospital in 95% of cases (Encuesta Nacional de Demografia y Salud, 2010). Although the maternal mortality rate has been falling, it is still far from goal at 72.9 per 100,000 live births, the leading cause of death is hypertensive disease of pregnancy (21%), followed by obstetric complications (14%), and maternal sepsis (7%) (DANE, 2009).

Genetic testing in Colombia is not covered by the mandatory health plan (POS, Plan Obligatorio de Salud) but patients can demand testing through legal action for protection of the right to health and life that must be solved expeditiously by judges against the health insurer (EPS), which will eventually be reimbursed by the government. Referrals to see a Clinical Geneticist are only covered by some EPS.

Although Colombia does not currently count with a rare disease program, work is being done in establishing one. The current census for patients with rare diseases is 13,168. A recently passed law (Law 1392 of 2010) enshrines guarantees of medical care and social protection for individuals with an orphan disease. The definition of orphan disease in the Colombian legislature is a medical condition with prevalence lower than 1 in 2,000 (Corte Constitucional de Colombia). There is a list of all conditions considered rare, ultra-rare and orphan in Colombia, the list is updated every 2 years (Ministerio de Salud y Proteccion Social, http://www.cuentadealtocosto.org/index.html: http://www.cuentadealtocosto.org/patologias/HUERFANAS/docs/generales/Resolucion-0430-2013.pdf).

## Genetic Disorders in Colombia

**Figure 3:**
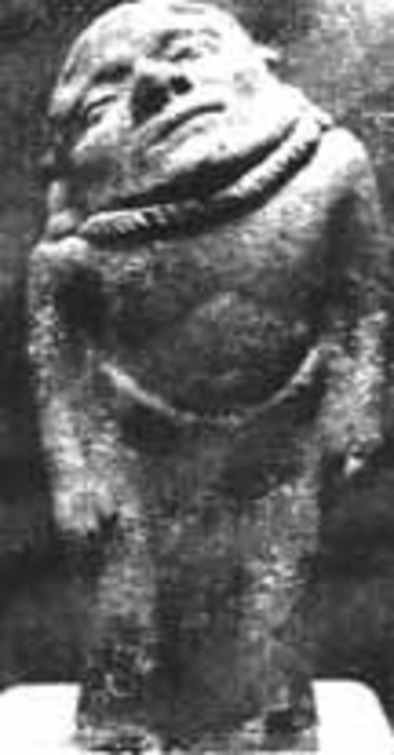
Tumaco-La Tolita clay figurine purportedly showing a patient with Morquio syndrome (Source: Museo Del Oro, Colombia).

We have evidence from the pre-Columbian era on the recognition of certain disorders from the Tumaco-La Tolita culture, a group of Amerindians settled in what is now the Colombian and Ecuador coast circa the year 600 bc. A collection of clay figurines have preserved in incredible detail the representation of what are thought to be prevalent genetic syndromes in the population at that time, such as: Morquio, Down syndrome, and Treacher-Collins (Museo del Oro) (Fig.3).

Colombia shows a characteristic pattern of prevalence of certain disorders based on ethnic distribution of some of its population, high prevalence of consanguinity and geographic isolation (the Andes Mountains have limited mobility of certain populations and isolated them to certain regions). The regions of Boyacá, Santander, and Antioquia see a higher incidence of several autosomal recessive conditions due to the high degree of consanguinity in rural regions. The prevalence of consanguinity in the country is the third highest in South America after Brazil and Venezuela at 1.30%, the majority of these mattings are between first cousins (Liascovich et al. 2001). No discussion on the effects of inbreeding in Colombia would be complete without referencing the work of Gabriel García Márquez. In his seminal book “Cien años de Soledad (One hundred years of solitude)”, Gabo (an affectionate nickname) writes about a large, multigenerational pedigree, that of the Buendía family in the fictitious town of Macondo (Márquez 1967). The themes of geographic isolation, inbreeding and incest are pervasive throughout the novel and weigh heavily in the mind of the author. Mental illness, fetal demise, congenital anomalies, dysmorphic features, and intellectual disability are all attributed in the novel as a direct or indirect consequence of consanguineous mating. The book ends describing the birth of a child with a caudal appendage (“pigtail” in the novel) product of a consanguineous mating between nephew and aunt bringing with it the end of the lineage (Castilla and Adams 1996).

Not many epidemiological studies have been carried out calculating the prevalence or cataloging the number of genetic conditions in Colombia, but data exist on conditions that are considered to be present in higher numbers within the Colombian population. Off the northwest coast of Colombia, the island of Providencia has a higher than average prevalence of sensorineural deafness, in general nonsyndromic deafness and in particular Waardenburg syndrome. The prevalence on the island is five cases per 1000, of which 65% are considered genetic. This is thought to be due to a founder effect. (Instituto Colombiano de Cultura Hispanica: http://www.banrepcultural.org/blaavirtual/geografia/geofraf1/indice.htm). Colombian investigators initially described Roberts's syndrome, a rare disorder characterized by severe limb and facial abnormalities after noticing an unusually high number of cases outside Bogota (Vega et al. 2005). These individuals shared a common ancestor in the 18th century and were pivotal in finding the causal gene, *ESCO2*. Since then several other cases have ben described, there are thought to be ∽150 cases worldwide, several of them in Colombia.

Huntington's disease is endemic in a small town in the northwestern part of the country, in the state of Atlántico; the town of Juan de Acosta contains the second largest population of patients with Huntington's disease in the world. The condition can be traced back to one of its founders, Lucas Echeverria who emigrated from Spain in 1790, generations of consanguineous mattings lead to the extensive number of affected members with the condition (El Espectador: http://www.elespectador.com/noticias/salud/enfermedad-rara-mas-comun-juan-de-acosta-articulo-478109). There are over 25 multigenerational families affected. Lastly, In Valle del Cauca, there is a cluster of 200 children affected with an autosomal recessive form of vitamin D-dependent rickets (Giraldo et al. 1995).

It is important to comment on a well-characterized and studied population within Colombia, that of the Paisas in the Antioquia region. As mentioned before, this community has a higher incidence of genetic disease owing to its geographical isolation and higher degree of consanguinity. The Paisas arose from several founder families, their genomes shaped by the admixture of Amerindian women and Caucasian (Spanish) men (Arcos-Burgos and Muenke 2002). This makes the Paisa population an excellent target as a genetic isolate for linkage and genome-wide association studies (GWAS); such studies have been carried out for a variety of conditions, such as ADHD (Arcos-Burgos et al. 2010), bipolar disorder (Kremeyer et al. 2010), Tourette syndrome (Scharf et al. 2013), benign hereditary chorea (Perez-Poveda et al. 2005), and facial clefting (Camargo et al. 2012). One of the most severe forms of early onset Alzheimer can be seen in this population due to a founder mutation, p.Glu280Ala in the *PSEN1* gene (Acosta-Baena et al. 2011). Other conditions described in the Paisas with a strong genetic component are multiple sclerosis, rheumatoid arthritis, Sjogren syndrome, and schizophrenia (Arcos-Burgos and Muenke 2002).

Other groups have carried out studies to calculate the prevalence of genetic disorders but these are far fewer and do not represent a concerted effort, for instance, the prevalence of mucopolysaccharidosis in Colombia is around two cases per 100,000, with type IV (Morquio syndrome) being the most common and type VII (Sly) being the least frequent (Gomez et al. 2012); the allele frequency for hemoglobin S in Choco region (predominantly Afro-Colombian) is 0.5% and 0.15% in Valle Del Cauca (Instituto Colombiano de Cultura Hispanica). The prevalence of cystic fibrosis in Colombia is thought to be higher than one in 12,000 although the authors of the study acknowledge that this is likely an underestimate given the high percentage of undiagnosed cases, significant delay in diagnosis (4 years average in Colombia) and early mortality of patients with CF (average range in Colombia is 2–25) (Vasquez et al. 2010).

Studies looking at the rate of congenital malformations and chromosomal abnormalities are even more limited. A study performed in Bogota and Cali, two of the biggest cities in the country, showed that the congenital malformation rate was 2.08%. Of these, cardiac malformations (13%), poly/syndactyly (10%), and multiple congenital anomalies (8%) were the three most common disorders (Garcia et al. 2014). Data from the ECLAMC initiative (Latin-American Collaborative Study of Congenital Malformations)-VIDEMCO group (Epidemiological Vigilance of Congenital malformations in Colombia) showed that the percentage of live birth infants with major malformations was 1.9% and the percentage of stillbirths with major malformations was 10% (Nazer and Cifuentes 2011). The same study showed that the most common malformations detected in Colombia among live birth infants were as follows: polydactyly (36%), club feet (24%), congenital cardiomyopathy (16%), hydrocephalus (13%), and cleft lip (13%). Of mention, there was a cluster of sirenomelia and cyclopia cases in Cali in 2008; in total, there were eight cases in a 165-day period. The cases were suspected of being related to heavy metal exposure for families that live downstream from the municipal landfill (Castilla et al. 2008). Microtia carries a similar prevalence as in other countries; most of the documented cases of microtia in Colombia are grade II and are associated with low birth weight. (Garcia-Reyes et al. 2009)

## Genetics Services in Colombia

Genetic services in Colombia are rendered in the great majority of cases by Academic institutions in the main cities: Bogota, Medellin, Cali, and Barranquilla.

Colombia has a state laboratory but it is limited in funding and resources, most if not all testing is performed in private laboratories (according to the Colombian Association of Human Genetics, there were at least 17 laboratories able to perform clinical molecular testing, and 12 laboratories able to perform cytogenetic testing), most of the laboratories doing genetic testing are affiliated with an Academic institution or are private for-profit companies. Testing is comprehensive and includes a variety of molecular techniques to include next-generation sequencing, preimplantation genetic diagnosis, and pharmacogenomics. Cytogenetic laboratories offer karyotypes, FISH, and chromosomal microarrays. Biochemical laboratories offer plasma amino acids, acylcarnitine profile, urine organic acids, and extensive menus of specialized biochemical testing.

There are organized efforts by patients for some genetic conditions, for instance, there is a Colombian Foundation for Cystic Fibrosis (https://es-es.facebook.com/FundacionColombianaParaFibrosisQuistica), which helps patients obtain appropriate care and treatment; the Colombian league for hemophiliacs (http://colhemofilicos.org.co); the association for lysosomal storage disorders (http://www.acopel.org.co/web/), Huntington's disease (http://fuhcol.blogspot.com/), and cleft lip and palate. There are also associations for individuals with congenital blindness, congenital deafness, and NF1.

Colombia has formal genetics training for medical professionals (physicians) in the form of a residency program that lasts 3 years; there are also master level courses that basic scientists can take. Physicians who complete a training program are boarded as Clinical geneticists. Genetics is also part of the medical school curriculum and students at the major academic institutions get exposure to a variety of patients with genetic conditions. There are no genetic counselors (GC) training in Colombia, the clinical geneticists, pediatricians, or Obstetricians/gynecologists do all counseling. The Colombian Society of Genetics is tasked with advancing the field, they host a conference every 2 years; trainees, resident, and faculty are encouraged to submit their work in the form of abstracts and lectures.

## Reproductive Law and Newborn Screening

Abortion is illegal in Colombia except in certain conditions: (1) In cases of rape; (2) When the pregnancy poses a health risk to the mother, or (3) Severe congenital malformation. Reform to reproductive rights in Colombia was a consequence of a high profile case in the country, that of Marta Gonzales. While pregnant Ms. Gonzales was diagnosed with cervical cancer, radiotherapy was denied since it would terminate the pregnancy. Ms. Gonzales eventually delivered a healthy baby girl but the cancer had metastasized, she died less than 2 years later. The case served as a flashpoint for reproductive rights (Sentence C-355 of 2006, Colombian Constitutional Court).

Colombia started a pilot newborn screening program in 1986 for congenital hypothyroidism (Carrillo, 1986), only more recently (1999) have efforts to institute a more broad NBS program have taken place. The current coverage is ∽36% with most samples coming from cord bloods. The program is decentralized and funded by three separate components of the health care system, as part of the mandatory program, only congenital hypothyroidism is screened for, other conditions offered on request include: PKU, galactosemia, congenital adrenal hyperplasia, biotinidase deficiency, hemoglobinopathies, and fatty acid oxidation disorders (Borrajo 2007).

More extensive NBS panels similar to ones offered in the United States can be obtained through in-house testing at private laboratories, although this testing is expensive and thus out of reach for most of the population.

## Final Remarks

The field of medical genetics is growing in Colombia; this is represented in the growing numbers of clinical geneticists, the wider variety of services, and the increasing availability and complexity of genetic testing offered to the public. It is encouraging that there are ongoing efforts to care and protect individuals with rare and orphan conditions, including expensive treatments that these patients would otherwise not be able to cover. The country still needs to address the delivery of care to underserved populations, work on increasing the number of newborns screened for inborn errors of metabolism, and the number of conditions screened for, and lastly increase the number of nonclinician genetics professionals. The rate-limiting step for many of these goals will be the availability of government funding.

## Conflict of Interest

None declared.
